# Sampling strategies to measure the prevalence of common recurrent infections in longitudinal studies

**DOI:** 10.1186/1742-7622-7-5

**Published:** 2010-08-03

**Authors:** Wolf-Peter Schmidt, Bernd Genser, Mauricio L Barreto, Thomas Clasen, Stephen P Luby, Sandy Cairncross, Zaid Chalabi

**Affiliations:** 1 Department of Infectious and Tropical Diseases, London School of Hygiene and Tropical Medicine, UK; 2 Instituto de Saúde Coletiva, Federal University of Bahia, Salvador, Brazil; 3 International Centre for Diarrhoeal Disease Research Bangladesh, Dhaka, Bangladesh; 4 Department of Public Health and Policy, London School of Hygiene and Tropical Medicine, UK

## Abstract

**Background:**

Measuring recurrent infections such as diarrhoea or respiratory infections in epidemiological studies is a methodological challenge. Problems in measuring the incidence of recurrent infections include the episode definition, recall error, and the logistics of close follow up. Longitudinal prevalence (LP), the proportion-of-time-ill estimated by repeated prevalence measurements, is an alternative measure to incidence of recurrent infections. In contrast to incidence which usually requires continuous sampling, LP can be measured at intervals. This study explored how many more participants are needed for infrequent sampling to achieve the same study power as frequent sampling.

**Methods:**

We developed a set of four empirical simulation models representing low and high risk settings with short or long episode durations. The model was used to evaluate different sampling strategies with different assumptions on recall period and recall error.

**Results:**

The model identified three major factors that influence sampling strategies: (1) the clustering of episodes in individuals; (2) the duration of episodes; (3) the positive correlation between an individual's disease incidence and episode duration. Intermittent sampling (e.g. 12 times per year) often requires only a slightly larger sample size compared to continuous sampling, especially in cluster-randomized trials. The collection of period prevalence data can lead to highly biased effect estimates if the exposure variable is associated with episode duration. To maximize study power, recall periods of 3 to 7 days may be preferable over shorter periods, even if this leads to inaccuracy in the prevalence estimates.

**Conclusion:**

Choosing the optimal approach to measure recurrent infections in epidemiological studies depends on the setting, the study objectives, study design and budget constraints. Sampling at intervals can contribute to making epidemiological studies and trials more efficient, valid and cost-effective.

## Introduction

The prevalence of common recurrent infections such as diarrhoea and respiratory infections in field studies is commonly estimated using repeated measurements in the same individuals. Many studies have used intensive surveillance, for example by conducting twice-weekly home visits to measure prevalence on every single day over the study period [1,2]. In other studies prevalence was measured at intervals, for example during only four home visits at 4-week intervals [3]. The differences in logistical effort are considerable. A study of 100 households over one year with twice-weekly surveillance visits would require 52 × 2 × 100 = 10,400 visits. Conducting only four visits per household in total requires only 4 × 100 = 400 visits. It has been shown that close surveillance can be inefficient with regard to study power [4]. To facilitate logistics and limit the impact of study procedures on participants' risk behaviour, it can be preferable to sample less frequently and recruit a somewhat larger study population to offset the loss of power incurring with fewer measurements [4].

Using an empirical mathematical model this paper explores the question of how many more participants are needed for infrequent sampling to achieve the same study power as frequent sampling. Furthermore, we tested the effect of recall error, and whether the use of daily point prevalence data offers any advantages over weekly period prevalence data [3,5]. Recording weekly period prevalence [3,5] may be simpler, but is less precise. Finally we explored the implications of clustering at group level (e.g. household or village) for sampling strategies.

## Theoretical Considerations

Repeated prevalence measurements allow the calculation of the "longitudinal prevalence" (i.e. the proportion-of-time-ill), a measure that in the case of diarrhoea has been shown to correlate better with adverse outcomes than incidence [6,7]. The longitudinal prevalence (LP) of a disease in an individual is a continuous outcome that can take values between 0% (never diseased) and 100% (always diseased) [4,6]. For sufficiently large studies, standard formulae for the calculation of the required sample size for the comparison of two means (e.g. in a control and intervention arm) can be used, such as

where *n *is the sample size per arm, the term (0.84+1.96) corresponds to 80% power and p = 0.05, σ_1 _and σ_2 _are the standard deviations of the LP in the two groups,  is the mean longitudinal prevalence in the control arm, and *LPR *the ratio of the mean LP between intervention and control arm [8].

The standard deviation can also be expressed in terms of the "coefficient of variation" (CV), useful for sample size calculations based on limited data (see below).

The sample size calculation for studies using LP as an outcome is not straightforward because the assumed standard deviation of  critically depends on how disease is distributed between individuals. For many common recurrent infections disease prevalence is highly clustered in individuals [9]. The more the disease is concentrated in high risk individuals, the easier it is to predict an individual's future disease evolution based on previous measurements, thus limiting the gain in study power from many repeat measurements.

There are several epidemiological characteristics of common recurrent infections like diarrhoea and respiratory infections that increase the clustering of disease in individuals, i.e. increase the standard deviation of the longitudinal prevalence, thereby making individuals more "different" from each other [9]. Episode incidence is typically highly clustered in high risk individuals [9]. Also, individuals with more episodes tend to experience longer episodes than those with fewer episodes [9]. This also concentrates disease days in high risk individuals. These and other characteristics can be specified in a mathematical model allowing the comparison of different surveillance strategies with regard to study power under controlled conditions [4,9].

## Simulation Model

Our model simulates the occurrence of recurrent infections in a population of hypothetical individuals over 365 days. For a detailed description of the model see [9]. The models were implemented in *Stata *10. The model was parameterized by specifying three major characteristics disease distribution.

### 1. Episode incidence

The distribution of the number of episodes is commonly highly skewed, with a minority of individuals experiencing many episodes. In the model, this is reflected by assuming that the number of episodes in individuals follows a gamma distribution [9].

### 2. Episode duration

The duration of episodes of most infections is also highly skewed with most episodes lasting for only one or two days. In the model the episode durations are assumed also to follow a gamma distribution with different parameters [9].

### 3. Correlation between incidence and episode duration

Individuals experiencing many episodes have been observed to also suffer from longer episodes [9]. This was modeled by assuming a linear association between episode incidence and mean episode duration in an individual. (Technical note: To improve model fit, the duration of each episode generated in the model is further modified by being multiplied by a normally distributed adjustment factor that assigns at random to each subject the tendency to experience predominantly shorter or longer episodes. For further details see [9]).

## The dependence of study power on sampling frequency: epidemiological determinants

In a first step, we built a set of models simulating 20,000 individuals with increasingly complex assumptions on how disease is distributed over the follow-up period of 365 days, based on a stepwise parameterization of the model described above. The aim of these models (four in total, named models A to D) was to illustrate how the three different model parameters (episode incidence, episode duration and correlation between incidence and episode duration) affect the dependency of study power on sampling frequency.

The parameters for this set of models were estimated from a single field study from Brazil, testing the effect of Vitamin A on diarrhea in children under 5 [10]. As observed in the study, we assumed a 5% prevalence of diarrhea in models A to D. The differences between models A to D are described in the next section. After generating the models A to D based on the model parameters, we simulated different sampling frequencies ranging between daily sampling to sampling only once every 28 days. For simplicity, we assumed a 24 h recall period without recall error. We then calculated the longitudinal prevalence (proportion of time ill) in each individual, the mean and standard deviation of the LP of the simulated population, and finally the required sample size for the different sampling intervals based on the formula above. For illustration, we assumed a 20% reduction of LP in one arm (*LPR *= 0.8, this value was not critical to the models' output).

### Results model A to D

**Figure 1:**
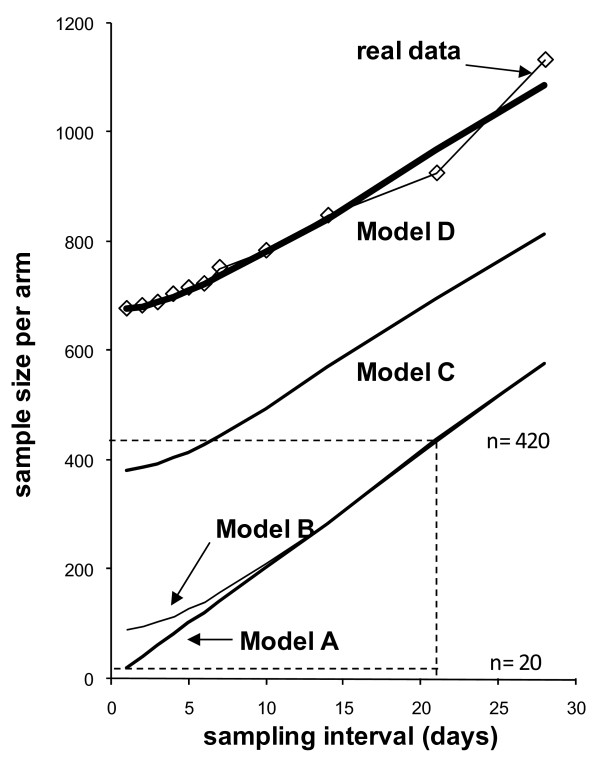
**Sample sizes per arm for different sampling intervals**. Longer intervals mean fewer simulated visits (sampling interval of 1 day means 365 visits, sampling interval of 28 days means 13 visits). *Model A *assumes that disease days are distributed completely at random; *Model B *assumes that diarrhoea occurs in episodes (as shown in Figure 3.2); *Model C *assumes (in addition to *B*) clustering of episodes in high risk individuals; *Model D *assumes (in addition to *C*) correlation between incidence and episode duration.

Model A is a simple (and unrealistic) model in which disease days (i.e. 5% of all days observed) are distributed completely at random between individuals and over the observation period (a Poisson process). As can be seen in Figure 1, sampling frequency and sample size in Model A are linearly related: For example, sampling only one day every 3 weeks (21 days) requires a 21 fold sample size (n = 420, dashed line) compared to daily sampling (n = 20, dashed line). In other words, it does not matter whether many measurements in few individuals or few measurements in many individuals are conducted: the study power only depends on the total number of visits.

**Figure 2:**
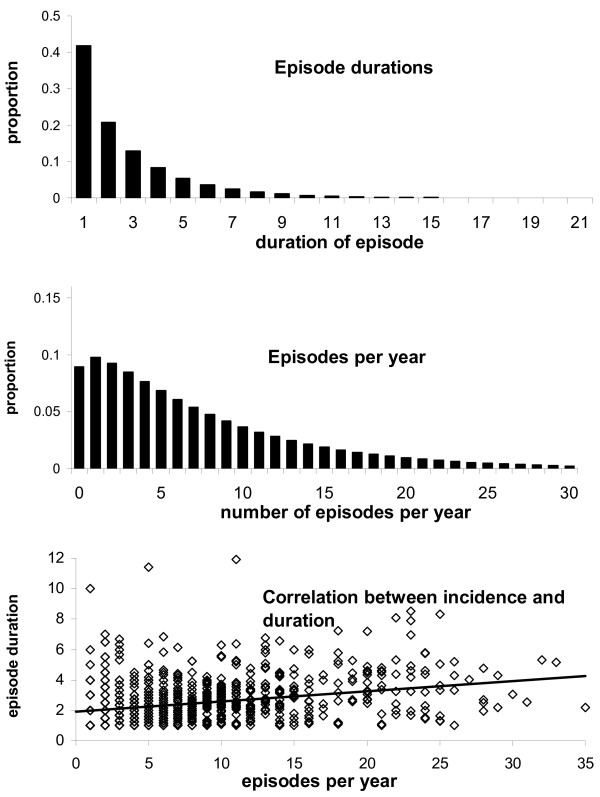
**Parameters for Models B, C and D based on diarrhea data from a Vitamin A trial in Brazil**. Top Panel: Assumed gamma distribution for episode duration with parameters α = 0.8 and β = 2.7. The observed and simulated mean duration of episodes was 2.7 days. Middle panel: assumed gamma distribution for the number of episodes with parameters α = 1.2 and β = 6.8. The observed and simulated mean number of episodes per year was 7.0. Bottom panel: Correlation between incidence and episode duration. Diamonds indicate the mean episode duration of individuals according to individual incidence. The line indicates the regression line with a slope corresponding to an increase of 0.07 days in episode duration with every additional episode an individual experiences.

Model B assumes that disease occurs in episodes of varying duration following a gamma distribution [9] whilst maintaining the percentage of days with illness in the whole population at 5% (Figure 2, top panel). In Model B, sampling every day requires nearly the same sample size as sampling every other day or every third day. However, for long sampling intervals the sample size converges to the linear relationship of Model A, where all disease days occur independently. Thus, due to the clustering of disease in episodes, sampling at 21 day intervals requires only a 4.8 fold increase in sample size compared to daily sampling (n = 89).

In Model C we assumed (in addition to gamma distributed episode durations) that the number of episodes per individual is drawn from a (different) gamma distribution, again with parameters estimated from the Brazil data (Figure 2, middle panel). The required sample size increases regardless of the sampling interval. The effect is that for sampling 1 in 21 days at regular intervals, the required sample size (n = 698) is only around 1.8 times larger compared to daily sampling (n = 382).

In Model D we assumed that the duration of episodes increases by 0.07 days with every additional episode, a value derived from linear regression analysis of the data from Brazil (Figure 2, bottom panel [9]). The required sample size increases for all sampling intervals, but slightly more for short intervals. Sampling 1 in 21 days at regular intervals now requires only a 1.4 times larger sample size (n = 969) compared to daily sampling (n = 674).

Figure 1 also shows the results if the sample sizes for different sampling intervals are estimated based on the real (instead of the simulated) data. The simulated data resulting from Model D and the real data produce very similar results, suggesting that the model incorporates the essential parameters for the purposes of this analysis. Further analysis revealed that adding seasonal variation [9] and autocorrelation (the dependency of disease risk on previous episodes [9,11,12]) hardly affected model results and the estimated sample sizes.

## The sample size increase with fewer measurements in different epidemiological settings

### Development of model scenarios

**Figure 3:**
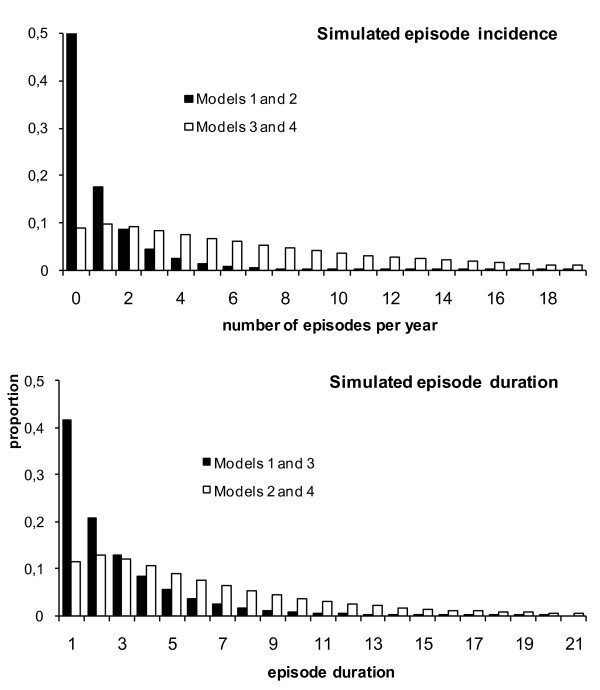
**Simulated distribution of the number of episodes per individual per year (top panel), and of the duration of episodes assumed for model scenarios 1 to 4 (bottom panel)**. The models scenarios are derived from a combination of the two different distributions for incidence and episode duration. Model scenario 1 combines low incidence and short duration, scenario 2 low incidence and long duration, scenario 3 high incidence and short duration, and scenario 4 high incidence and long duration. The black bars in top panel show the distribution assumed for the two scenarios with a low incidence, the white bars the distributions for the models with high incidence. Similarly, the black bars in bottom panel show the distribution of episode durations for the two models with a short mean episode duration, the white bars distribution for the models with long mean duration.

The above findings illustrated the importance of disease distribution within and between individuals on the choice of the required sampling intervals. In a second step we developed a set of four model scenarios using parameters from a range of field studies across the world [9] with the aim of quantifying the association between sampling frequency and required sample size under more realistic model assumptions. The model scenarios were developed to cover a broad range of epidemiological settings with a focus on diarrhoea and respiratory infections. They were derived from the combination of two different distributions of disease incidence ('low risk' and 'high risk'), and two different distributions of episode duration ('short' duration and 'long' duration). The model scenarios are described in Table 1 and Figure 3.

**Table 1:** Four model scenarios with examples

	Short episode duration	Long episode duration
**Low incidence**	**Model scenario 1 (LS)**	**Model scenario 2 (LL)**
		
	**Annual incidence: **0.9/person-year	**Annual incidence: **0.9/person-year
	**Mean Episode duration: **2.7 days	**Mean Episode duration: **5.6 days
		
	*Examples:*	*Examples:*
	-Diarrhea or fever in low risk child population (e.g. Thailand [27])	-ALRI in malnourished child populations (Ghana[14], Brazil 2[10])
		-Diarrhoea in an population with a very heterogeneous risk (e.g. Guatemala [13])

**High incidence**	**Model scenario 3 (HS)**	**Model scenario 4 (HL)**
		
	**Annual incidence: **7.0/person-year	**Annual incidence: **7.0/person-year
	**Mean Episode duration: **2.7 days	**Mean Episode duration: **5.6 days
		
	*Examples:*	*Examples:*
	-Diarrhoea or fever in high risk child populations, like Brazil[10], Peru[28]	-Diarrhoea in very poor settings in undernourished children, e.g. Ghana[14]
		-Mild ARI in high risk population (Ghana, Brazil 2[10,14])

Model scenario 1 ("**L**ow incidence/**S**hort duration"- **LS**) represents a population with fairly low risk of disease (0.9 episodes per person-year) of short duration (mean 2.7 days). Model scenario 2 ("**L**ow incidence/**L**ong duration" - **LL**) assumes the same incidence as model scenario 1, but with long illness duration (mean 5.6 days), suitable to represent relatively uncommon but severe repeated infections like acute lower respiratory infections. It may also be suitable to represent diarrhoea risk in a very heterogeneous population (in terms of age or socioeconomic status) where a small subset of the population experiences many episodes of long duration [13]. Model scenarios 3 and 4 represent diseases that occur at a very high incidence (7 episodes per person-year on average) with either short duration ("**H**igh incidence/**S**hort duration" - **HS**), e.g. diarrhoea in a trial in Brazil,[10], or long duration ("**H**igh incidence/**L**ong duration" - **HL**) like diarrhoea in a trial in Ghana [14] or cough in the Brazil trial [10].

The correlation between episode duration and individual incidence and the intra-subject correlation of episode durations were fitted to field data from Guatemala (Model scenarios 1 and 2) [13], Brazil (Model scenario 3) [10] and Ghana (Model scenario 4)[14].

### Simulated surveillance strategies

For the four different model scenarios we simulated surveillance visits at varying intervals over the simulated 365 days. At each simulated visit, we applied different recall periods to simulate four commonly used recall approaches:

(1) Point prevalence over the last 24 hours ("Did you have the disease during the last 24 hours?").

(2) Point prevalence for the last 3 days ("On which of the last 3 days did you have the disease?")

(3) Point prevalence for the last 7 days ("On which of the last 7 days did you have the disease?").

(4) Period prevalence over the last seven days ("Did you have the disease at any time during the last 7 days?").

It has been shown that recall periods longer than 48 hours are prone to recall error [15-17]. Based on published data, we assumed that disease on the 48 h before a simulated visit is always reported, while disease on day -3 to -7 prior to a visit is reported with a probability of 0.74, 0.67, 0.67, 0.58 and 0.58, respectively [15]. All results were averaged over 100 simulation runs which were found to be sufficient to achieve robust estimates.

### Simulation results

Table 2 shows the estimates of LP (and SD) obtained by applying the four surveillance strategies to the four model scenarios reflecting different assumptions of the underlying disease distribution. This table only shows the results for 52 (= weekly) visits over the simulated period of one year. The true mean LP in the control group is 0.6% in model scenario 1 (LS), 1.3% in model scenario 2 (LL), 5.4% in model scenario 3 (HS) and 11.0% in model scenario 4 (HL). Since we assumed 100% accuracy for the 24 h recall period, the mean LP resulting from applying 24 h recall at 52 visits over one year (52 days sampled per individual at 1 week intervals) are an unbiased estimate of the true mean LP in the simulated populations. For the other recall periods we assumed recall error. Some days of illness are 'forgotten', leading to smaller estimates of the mean LP (Table 2).

**Table 2:** LP, standard deviation, and LP ratios resulting from different recall periods

	control group		intervention group		
	
Model/recall method	mean LP	SD	mean LP	SD	LP ratio
*Model scenario 1 (LS)*					
1 day point prevalence recall	0.6%	1.5%	0.5%	1.2%	0.80
3 day point prevalence recall	0.5%	1.3%	0.4%	1.1%	0.80
7 day point prevalence recall	0.4%	1.0%	0.3%	0.8%	0.80
7 day period prevalence recall	1.6%	3.2%	1.3%	2.7%	0.81

*Model scenario 2 (LL)*					
1 day point prevalence recall	1.3%	3.2%	1.0%	2.7%	0.80
3 day point prevalence recall	1.2%	2.9%	0.9%	2.4%	0.80
7 day point prevalence recall	1.0%	2.4%	0.8%	2.0%	0.80
7 day period prevalence recall	2.4%	5.0%	1.9%	4.2%	0.82

*Model scenario 3 (HS)*					
1 day point prevalence recall	5.4%	7.3%	4.2%	5.9%	0.80
3 day point prevalence recall	4.9%	6.5%	3.9%	5.2%	0.80
7 day point prevalence recall	4.0%	5.2%	3.2%	4.2%	0.80
7 day period prevalence recall	13.9%	13.9%	11.5%	11.8%	0.84

*Model scenario 4 (HL)*					
1 day point prevalence recall	11.0%	13.1%	8.9%	11.1%	0.80
3 day point prevalence recall	10.1%	11.9%	8.1%	10.1%	0.80
7 day point prevalence recall	8.2%	9.7%	6.6%	8.2%	0.80
7 day period prevalence recall	19.3%	18.7%	16.0%	16.3%	0.82

Simulated were 2000 individuals allocated to intervention and control group, with a 20% LP reduction in the intervention group (LP ratio= 0.8).

Using weekly *period *prevalence data (summing up the disease experience over one week) results unsurprisingly in larger mean LP estimates and a larger SD of the mean LP. However, the CV, i.e. the SD divided by the mean LP, is smaller for period prevalence data than for point prevalence data, which has consequences for the study power. For example, in the case of model scenario 1, the CV for using 7-day-recall *point *prevalence data is 1%/0.4%= 2.5, but is only 3.2%/1.6%= 2 for recording 7-day-recall *period *prevalence. Thus, recording period prevalence reduces the differences in LP between study participants. It is easy to see why: individuals who had diarrhoea at some point during the last 7 days may have suffered from one or more episodes of different duration. The number of diarrhoea days in the last seven days in these individuals may be anything between one and seven, but when period prevalence data are recorded they are all simply coded as "diseased at any time during the last 7 days". Applying the mean LP and standard deviations derived from period prevalence data to the sample size formula therefore may result in lower sample size estimates compared to daily point prevalence data (see below).

The simulations identified a slight bias in the estimate of the risk ratio introduced by the use of period prevalence data (Table 2). While all point prevalence estimates (regardless of recall error) result in unbiased risk ratios, using period prevalence biases the risk ratio towards one. Bias is strongest for the "high risk/short duration" model scenario 3, with the risk reduction being biased from -20% to -16% (RR= 0.84, Table 2). The bias is due to the possibility that some individuals in the control group suffer from two or more episodes in a given week of observation while some individuals in the intervention group only suffer from one episode during that week (due to the effect of the intervention). These individuals are all coded as "diseased" during that week if period prevalence data are used, thus reducing the differences between the two groups.

**Figure 4:**
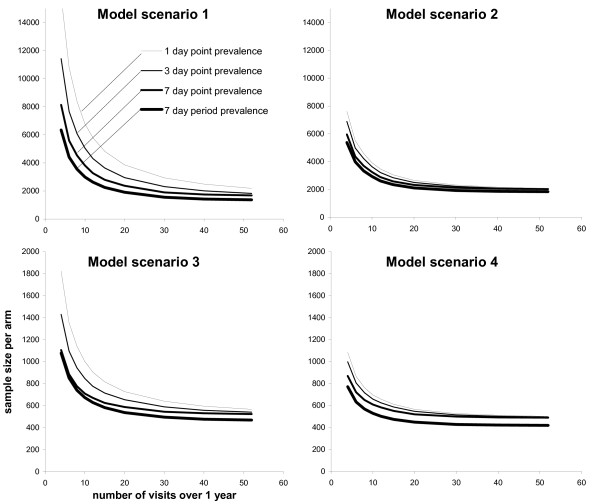
**Sample size for the comparison of two groups (20% LP reduction in one arm, 80% power, p = 0.05) as a function of the number of surveillance visits over one year for the 4 different model scenarios**. For each model scenario, the 4 lines show the sample size assuming the 4 different recall strategies.

Figure 4 shows the effect of varying the number of household visits (X-axis) on the estimated sample size (Y-axis) of a study comparing two groups with a 20% LP reduction in one arm (80% power and p = 0.05). For each of the four model scenarios, the different lines show the simulation of the four different recall approaches applied (24 h, 3 days and 7 days point prevalence; one week period prevalence). As can be expected, short recall periods (24 h or 3 days) require the highest sample sizes, especially for a small number of visits.

In general the simulations imply that 52 visits over one year are always inefficient. Conducting 20 visits instead of 52 only requires a marginally larger sample size to achieve the same study power, regardless of the recall period chosen. Increasingly larger sample sizes are needed for fewer visits, although the increase can be lowered by using long recall periods (Figure 4).

Perhaps counter-intuitively, all model scenarios, but in particular the two high risk model scenarios 3 (HS) and 4 (HL), show that recording one-week period prevalence is more efficient in terms of study power than the more informative one-week point prevalence data. As discussed above, this finding is due to the smaller CV that results from collecting period prevalence data compared to point prevalence data (Table 2). The effect of the smaller CV overrides the increase of the sample size resulting from a slightly biased risk ratio. Thus, period prevalence data provide a more precise but slightly biased estimate of the prevalence reduction.

Table 3 shows the association between sampling frequency and sample size, expressed as multiplication factors indicating the increase in the sample size relative to that for 52 visits. For example, a study using 7 day point prevalence in situations similar to model scenario 3 (HS) will require a 30% larger sample size if 12 instead of 52 visits are conducted. On the other hand, a 90% larger sample size is required if 12 instead of 52 visits are conducted for situations corresponding to the low risk scenario 1 (LS).

**Table 3:** Multiplication factors for the required sample sizes

	52 visits	20 visits	12 visits	6 visits	4 visits
*Model scenario 1 (LS)*					
7 day point prevalence recall	1.0	1.4	1.9	3.3	4.8
3 day point prevalence recall	1.0	1.6	2.4	4.2	6.3
7 day period prevalence recall	1.0	1.4	1.9	3.2	4.6
Incidence during 7 day recall	1.0	1.6	2.2	3.7	5.2
					
*Model scenario 2 (LL)*					
7 day point prevalence recall	1.0	1.1	1.4	2.2	2.9
3 day point prevalence recall	1.0	1.2	1.6	2.4	3.4
7 day period prevalence recall	1.0	1.1	1.4	2.2	2.9
Incidence during 7 day recall	1.0	1.5	2.1	3.7	5.1
					
*Model scenario 3 (HS)*					
7 day point prevalence recall	1.0	1.1	1.3	1.7	2.1
3 day point prevalence recall	1.0	1.2	1.4	2.0	2.6
7 day period prevalence recall	1.0	1.1	1.3	1.8	2.3
Incidence during 7 day recall	1.0	1.3	1.7	2.5	3.3
					
*Model 4 scenario (HL)*					
7 day point prevalence recall	1.0	1.1	1.2	1.5	1.8
3 day point prevalence recall	1.0	1.1	1.3	1.6	2.0
7 day period prevalence recall	1.0	1.1	1.2	1.5	1.8
Incidence during 7 day recall	1.0	1.4	1.8	2.7	3.7

Multiplication factors for the required sample size to achieve the same statistical power as a study using weekly disease sampling over one year (52 visits). Values are given for 4 different surveillance strategies (in addition to the baseline strategy) to measure disease prevalence applied to the four different model scenarios. For comparison, these values are also calculated for using incidence instead of prevalence. Incidence was defined as the occurrence of any new episode during the 7 day recall period with a gap of at least 2 days between two new episodes. The assumptions on recall error were the same as for prevalence measures (but these did not impact on the model results, see sensitivity analysis).

We further tested whether disease sampling at intervals may also be applicable to studies measuring the incidence of infection rather than LP. Here we defined incidence as any new episode occurring within the 7-day recall period, with a gap of two days between diarrhoea days (commonly required to define a new episode). The results are given in Table 3. In all scenarios, decreasing the number of visits leads to a larger sample size increase for incidence than for LP.

### Group-level clustering

Many diarrhoea studies need to consider clustering of disease in households, villages or other groups, for example in situations where an intervention is randomised at group level. The degree of clustering can be described as the intra-cluster correlation coefficient (*ICC*) which can calculated as follows [8]:

where *σ_B _*is the between-cluster standard deviation and *σ_W _*the within-cluster standard deviation. With fewer measurements per individual, the within-cluster standard deviation of the LP increases, because the individual LP estimates are less precise. As a consequence, the *ICC *will decrease. The *ICC *can be used to calculate the design effect *Deff*, the factor by which the sample size of a study needs to be inflated to account for clustering [8]:

where *m *is the number of individuals per cluster. As the *ICC *decreases with less frequent sampling, *Deff *also decreases. As a consequence, the sample size inflation due to clustering is smaller for infrequent sampling than for frequent sampling.

We incorporated group-level clustering by allocating the simulated individuals to clusters assuming approximately normally distributed mean cluster prevalences. For illustration we tested cluster sizes of n = 5 (to represent households) and n = 50 (to represent villages). We assumed *ICC *values based on published data [18-20]. We tested *ICC *values of 0.04, 0.1 and 0.3 for a cluster size of n = 5, and *ICC *values of 0.005, 0.02 and 0.1 for a cluster size of n = 50. The *ICC *was estimated using the Stata command *loneway*. For the *ICC *calculation, longitudinal prevalence was treated as a continuous outcome measure.

The effect of group level clustering on the sample size inflation factors (Table 3) is shown in Table 4. For simplicity, we only investigated surveillance using 7 day recall of daily point prevalence. In general, intra-class correlation of disease reduces the sample size multiplication factors. For example, assuming an *ICC *of 0.02 and a cluster size of n = 50 in model scenario 3, the sample size increase will only be 20% instead of 30% if sampling frequency is reduced from 52 to 12 visits.

**Table 4:** Multiplication factors for the required sample sizes accounting for clustering at group level

	52 visits	20 visits	12 visits	6 visits	4 visits
***Model scenario 1 (LS)***					
no clustering	1.0	1.4	1.9	3.3	4.8
Cluster size n = 5					
*ICC *= 0.04	1.0	1.4	1.8	3.0	4.3
*ICC *= 0.1	1.0	1.3	1.7	2.7	3.8
*ICC *= 0.3	1.0	1.2	1.4	2.1	2.8
Cluster size n = 50					
*ICC *= 0.005	1.0	1.3	1.8	2.9	4.2
*ICC *= 0.02	1.0	1.2	1.5	2.2	3.0
*ICC *= 0.1	1.0	1.1	1.2	1.4	1.7

***Model scenario 2 (LL)***					
no clustering	1.0	1.1	1.4	2.2	2.9
Cluster size n = 5					
*ICC *= 0.04	1.0	1.1	1.4	2.0	2.7
*ICC *= 0.1	1.0	1.1	1.3	1.8	2.4
*ICC *= 0.3	1.0	1.1	1.2	1.5	1.9
Cluster size n = 50					
*ICC *= 0.005	1.0	1.1	1.3	1.9	2.6
*ICC *= 0.02	1.0	1.1	1.2	1.6	2.0
*ICC *= 0.1	1.0	1.0	1.1	1.2	1.4

***Model scenario 3 (HS)***					
no clustering	1.0	1.1	1.3	1.7	2.1
Cluster size n = 5					
*ICC *= 0.04	1.0	1.1	1.2	1.6	2.0
*ICC *= 0.1	1.0	1.1	1.2	1.5	1.8
*ICC *= 0.3	1.0	1.1	1.1	1.3	1.5
Cluster size n = 50					
*ICC *= 0.005	1.0	1.1	1.2	1.6	1.9
*ICC *= 0.02	1.0	1.1	1.2	1.4	1.6
*ICC *= 0.1	1.0	1.0	1.1	1.2	1.3

***Model scenario 4 (HL)***					
no clustering	1.0	1.1	1.2	1.5	1.8
Cluster size n = 5					
*ICC *= 0.04	1.0	1.1	1.2	1.4	1.7
*ICC *= 0.1	1.0	1.0	1.1	1.3	1.6
*ICC *= 0.3	1.0	1.0	1.1	1.2	1.4
Cluster size n = 50					
*ICC *= 0.005	1.0	1.1	1.2	1.4	1.6
*ICC *= 0.02	1.0	1.0	1.1	1.2	1.4
*ICC *= 0.1	1.0	1.0	1.0	1.1	1.2

The sample size is calculated for a study comparing the mean LP between two groups with a 20% LP reduction in one arm (80% power and p = 0.05). Sampling was simulated as 7 day point prevalence recall at each visit (see Table 3.).

### Sensitivity analysis

We used model scenario 3 (HS, Table 1) as the default model scenario for the sensitivity analysis (the other scenarios showed similar findings). The results of the simulations were robust against reducing disease incidence in the intervention arm by lowering the α-parameter of the gamma distribution (which increases the skew of the distribution) instead of the β-parameter of the gamma distribution for incidence (not shown); α and β are respectively the shape and stretch parameters of the gamma distribution.

In a further analysis we assumed that the 20% reduction of LP occurs only through a reduction in the duration of episodes (by reducing the β-parameter of the gamma distribution for episode duration), while the incidence remains the same in both study arms. For the point prevalence data the sample sizes for the different surveillance intervals were similar to the default model scenario, where LP was reduced by decreasing incidence. In contrast, the use of weekly period prevalence data biased the LP ratio from the true value of 0.8 to 0.92 (a 20% LP reduction vs. a 8% LP reduction). This increased the required sample size by a factor of about 4 regardless of sampling frequency. Thus, for the study of interventions or risk factors affecting episode duration, the use of period prevalence data can result in strongly biased estimates towards no effect and low study power.

In the default model scenario (HS) we had assumed recall error according to published field data. These might overestimate recall error, since it is plausible that the higher diarrhoea prevalence closer to the surveillance visit sometimes may indicate that household members remember diarrhoea during the last seven days as having occurred more recently than was actually the case. Omitting recall error from the model scenario only slightly lowers the increase in the sample size for all sampling approaches. On the whole, the model results were not sensitive to the assumptions on recall error.

## Sample size calculation in practice

For the sample size calculation in practice, an investigator must first identify a reasonable estimate of the baseline mean LP in the population, which can be acquired from cohort or cross sectional data. The challenge lies in the determination of the standard deviation of LP which depends on factors that are often unknown, unless high quality longitudinal data are available. Field data [9] suggest that the ratio of the SD of LP to the mean LP (the CV) will decrease from model scenario 1 to 4. A range of CV values estimated from studies available to the authors are listed in Table 5. Typical CV values for model scenarios 1 (LS) and 2 (LL) range between 2.5 (Guatemala and Pakistan diarrhoea data) and 2.9 (Ghana ALRI data). For model scenarios 3 (HS) and 4 (HL) one may assume CVs of about 0.9 to 1.3 (Ghana diarrhoea and Brazil 2 diarrhoea data). These large differences in the standard deviations between the scenarios highlight the difficulty in estimating the required sample size.

**Table 5:** Observed standard deviations and coefficients of variation in different study populations

Study	Mean LP	SD	CV
Guatemala [13]			
Diarrhoea	0.023	0.057	2.45
Pakistan [29]			
Diarrhoea	0.014	0.038	2.65
Brazil 1 [2]			
diarrhoea	0.029	0.045	1.54
Brazil 2 [10]			
diarrhoea	0.050	0.066	1.31
cough	0.238	0.187	0.78
fever	0.042	0.040	0.95
Ghana [14]			
diarrhoea	0.170	0.171	1.01
cough	0.141	0.152	1.12
rapid breathing	0.014	0.042	2.87

LP and SD were calculated based on point prevalence data collected continuously over one year

### Example

Suppose an investigator wants to estimate the sample size for a one-year intervention trial to reduce the LP of diarrhoea by 20% in a child population with a known LP of 2.5% (80% power and p = 0.05). Based on limited epidemiological data from the site, the investigator assumes that the mean episode duration is fairly short (2 to 3 days) and the incidence between 4 to 6 episodes per child year [9]. This means that the episode duration is similar to model scenarios 1 and 3 (2.7 days), while the incidence is approximately between the values assumed for these two model scenarios (model scenario 1 = 0.9 and model scenario 2 = 7.0 per person-year). The CV may be around 1.6 which leads to a standard deviation in the control arm of 4.0. This results in a sample size of 823 per arm if all the days over the year were sampled. If the investigator decides to limit the number of visits to one per month, then in this case example, the increase should be between the factors for model scenarios 1 and 3, approximately around 1.5, which results in a sample size of n = 1235 per arm instead of n = 823.

## Discussion

Sampling at long intervals to measure the prevalence of common infections may not only reduce the number of visits and costs, but could also improve participants' willingness to cooperate and decrease the potential for changing risk behaviours due to close surveillance. The findings of this study could be used to inform on the choice of the most appropriate sampling strategy and its implications for sample size and sampling frequency. Infrequent sampling is less suitable when the aim of a study is to measure the incidence of infection.

Sampling strategies are ideally chosen to maximise study power given the available budget. The choice of a particular sampling strategy in terms of costs and logistics is highly context specific. In some settings, recruiting and supervising a large group of field workers (needed for intensive follow-up) can be straightforward, in other settings very difficult. Sometimes, recruiting additional study participants can be the dominant logistical challenge. In this case it may be better to choose close follow-up to maximise study power given a limited number of participants.

The final choice of the sampling frequency may often depend on the research question. If microbiological data are to be collected, frequent sampling may be necessary to maximize study power particularly for uncommon pathogens. Long sampling intervals may be ideal for example to explore the effect of large scale-environmental health interventions, where the causative pathogen is often of minor interest and the study population is too large to allow frequent sampling. But regardless of the study question, it should be useful to compare the statistical power of different sampling frequencies (for example by using Tables 3 and 4).

In the past, large-scale trials have often opted for close surveillance of a subset of the population [14]. In many circumstances it may be better to sample disease at long intervals in the whole study population to save staff costs and to avoid influencing risk and reporting behavior of study participants by frequent visits. A recent trial on water treatment in Kenya in which participants were randomized to two different diarrhoea surveillance schemes (intensive vs. infrequent sampling) found strong evidence for the latter (Michael Kremer, Claire Null, personal communication).

The following example illustrates the profound implications on study planning of choosing infrequent sampling. Emerson and colleagues conducted a large cluster-randomised trial to measure the impact of fly control and latrine construction on trachoma [21]. Diarrhoea was originally included as a secondary outcome but then dropped because the logistical effort of conducting weekly follow-up visits was deemed prohibitive (Emerson, personal communication). However, our results suggest that the additional costs of visiting each household 6 to 12 times over the study period of 6 months would have been small, and - given the large number of already recruited participants - could have allowed estimating the effect size of sanitation on diarrhoea with sufficient accuracy. Given the clustered design of the trial, close surveillance of participants for diarrhoea symptoms would have gained little power over less frequent sampling, as was shown in Table 4. Jenkins and colleagues conducted a study on the impact of a household water filter on diarrhoea [22]. Due to lack of funding, the study was originally planned as an acceptability study, only. However, after considering different surveillance strategies as described in this paper the budget was judged sufficient to conduct 6 visits per household at monthly intervals.

Some interventions or risk factors (e.g. micronutrient supplements) partly or primarily affect the duration rather than the incidence of infections [10]. Some interventions can also alter the average duration of disease by selectively reducing short or long episodes as observed in a household water treatment trial study in Guatemala [13]. Period prevalence data may at times be more efficient in terms of study power and are easier to collect. However, it will often be difficult to exclude prior to a study that an intervention affects episode duration, which would not be captured fully by recording period prevalence. The results indicate that in most circumstances, applying a 7-day recall period using point prevalence data may be the preferred choice for measuring prevalence. However, the choice of the length of the recall period depends on the situation. For example, in urban settings people may be used to Monday-Friday work weeks and shop opening hours and might 'think' in weekdays more than some poor rural populations, which may facilitate disease recall.

We identified several epidemiological characteristics of recurrent infections that needed to be included in the simulations in order to achieve estimates on the association between sampling frequency and sample size. A large number of parameter combinations would have been possible, and it could be argued that the simulation should have focused on changing these parameters individually. However, different settings commonly do not differ in a single parameter but in several of them jointly. For example, children in high incidence settings often have longer episodes than in low incidence settings [9]. We limited the number of scenarios to just four, covering a fairly wide range of epidemiological settings and conditions. As with any more complex model the choice of these scenarios was to some extent arbitrary. As in the above example of a sample size calculation many epidemiological settings will fall in between the scenarios, so that the sample size calculation will still contain a fair amount of guess-work. Future work could explore a wider range of model scenarios, including other types of infections and conditions, which in this analysis we largely restricted to diarrhoea and respiratory infections as the most important in terms of morbidity and mortality [23].

As demonstrated in Figure 1 the simulated datasets used in this analysis allowed us to explore the role of different parameters of disease distribution under "controlled conditions". Real datasets would have been unsuitable for this purpose because it would have been very difficult to infer from them a similar understanding of the stochastic processes that may influence the choice of the appropriate sampling strategies. However, simulation models by definition simplify the dynamics of disease occurrence and as such they provide an approximation of the real data. For example, the model does not allow for missing data, which occur in most datasets collected in the field. The validation of the model using the real datasets (which all contained missing data of up to 10%) revealed that missing data do not systematically influence the association between sampling frequency and sample size in studies with a typical loss-to-follow up (see the comparison of real and simulated data shown in Figure 1). For this reason, we did not further explore the complex issue of missing data.

As described in the sensitivity analysis section, our assumptions regarding recall error were relatively straightforward. For example, we assumed the same recall error for recording point prevalence and period prevalence data. Obtaining point prevalence data will require a more thorough questioning of study participants compared to period prevalence (which can be obtained with a single question). Spending more time with the interviewees may reduce recall error.

In real datasets, recall error may also vary between different villages/clusters and increase during the course of a study due to a number of factors. Towards the end period of a study, prevalence estimates are often low suggesting that participants or field staff lose interest in reporting disease [11]. Some studies found that the first (or a single) surveillance visit provides higher, at times implausible prevalence estimates compared to subsequent visits [24,25]. Our model did not account for these factors. However, the sensitivity analysis showed that recall error hardly influences the model results. Also, recall error could be less important for calculating LP than incidence of disease because the timing of disease occurrence is less relevant. A recent study found that mothers often misplaced the day at which disease occurred in a child, but this error had little effect on the overall prevalence estimate [26].

## Conclusions

Choosing the optimal approach to measure recurrent infections in epidemiological studies greatly depends on the setting, the study objectives, study design and budget constraints. Our findings may contribute to making epidemiological studies more efficient, valid and cost-effective. They may also encourage more researchers to include diarrhea or respiratory infections as a health outcome in the first place, which previously have often been thought to require a high logistical effort. As shown in this paper, this need not necessarily be the case.

## Competing interests

The authors declare that they have no competing interests.

## Authors' contributions

WPS designed the study, developed the model and lead on writing the manuscript. BG designed the study and contributed to the manuscript. ZC contributed to the design of the study and model development and contributed to the writing of the manuscript. MB, SL, TC and SC contributed to the development of the project, the study design and writing of the manuscript. All authors have read and approved the final manuscript.
